# Is machine translation a dim technology for its users? An eye tracking study

**DOI:** 10.3389/fpsyg.2023.1076379

**Published:** 2023-02-06

**Authors:** Ramunė Kasperė, Jurgita Motiejūnienė, Irena Patasienė, Martynas Patašius, Jolita Horbačauskienė

**Affiliations:** ^1^Faculty of Social Sciences, Arts and Humanities, Kaunas University of Technology, Kaunas, Lithuania; ^2^Faculty of Informatics, Kaunas University of Technology, Kaunas, Lithuania

**Keywords:** machine translation, acceptability, usability, quality, satisfaction, end-users, professional translators

## Abstract

State-of-the-art research shows that the impact of language technologies on public awareness and attitudes toward using machine translation has been changing. As machine translation acceptability is considered to be a multilayered concept, this paper employs criteria of usability, satisfaction and quality as components of acceptability measurement. The study seeks to determine whether there are any differences in the machine-translation acceptability between professional users, i.e., translators and language editors, and non-professional users, i.e., ordinary users of machine translation who use it for non-professional everyday purposes. The main research questions whether non-professional users process raw machine translation output in the same way as professional users and whether there is a difference in the processing of raw machine-translated output between users with different levels of machine-translated text acceptability are analyzed. The results of an eye tracking experiment, measuring fixation time, dwell time and glance count, indicate a difference between professional and non-professional users' cognitive processing and acceptability of machine translation output: translators and language editors spend more time overall reading the machine-translated texts, possibly because of their deeper critical awareness as well as professional attitude toward the text. In terms of acceptability overall, professional translators critically assess machine translation on all components of which confirms the findings of previous similar research. However, the study draws attention to non-professional users' lower awareness regarding machine translation quality. The study was conducted within a research project that received funding from the Research Council of Lithuania (LMTLT, agreement No S-MOD-21-2), seeking to explore and evaluate the impact on society of machine translation technological solutions.

## 1. Introduction

Neural machine translation is more and more frequently used in the translation and localization market. Following the AI Index Report, artificial intelligence has allowed improving machine translation in certain language pairs almost to human quality (Perrault et al., 2019). According to some scores, “[t]he fastest improvement was for Chinese-to-English, followed by English-to-German and Russian-to-English” (Perrault et al., 2019). However, the performance varies between different language pairs and that depends on language pair popularity, which “defines how much investment goes into data acquisition” (Perrault et al., 2019).

For these reasons, researchers and research administrators have recently been paying attention to the effects that artificial intelligence and developed technologies bring about on the translation industry, translator's profession, career and daily tasks, as well as training and skills needed, but also in terms of the perceptions within society. In this perspective, some important research papers have been published in the past few years where translation scholars have concluded that, for example, artificial intelligence-powered machine translation and other language related technologies have fundamentally changed public awareness and attitudes toward multilingual communication (Vieira et al., 2021). Such technologies are now increasingly being used to overcome language barriers not only in situations of personal use but also in high-risk environments, such as health care systems, courts, police and so on. The availability and impact of machine translation accessibility and impact on society, including the importance of full participation of various social groups in communication processes, are being analyzed and evaluated (Vieira et al., 2021). On the other hand, public awareness of the capabilities as well as the quality of machine translation is identified as insufficient (Kasperė and Motiejūnienė, 2021).

Although machine translation is breaking down language barriers, and its accuracy and efficiency are getting closer to human-level translation, human effort is needed to reduce the negative impact of machine translation in society (Hoi, 2020). The communication processes supported by machine translation can be of high quality if the process participants are aware of the quality shortcomings (Yasuoka and Bjorn, 2011). Studies have also found that machine translation can help reduce the exclusion of ethnic minorities in a wide variety of fields (Taylor et al., 2015).

There is a plethora of research on the quality of machine translation and use of post editing (see Ueffing, 2018; Ortega et al., 2019; Vardaro et al., 2019; Nurminen and Koponen, 2020; Rossi and Carré, 2022, to mention but a few). The benefits of machine translation post editing in different language pairs have been acknowledged in multiple studies employing a diversity of research designs (see Carl et al., 2011, 2015; Moorkens, 2018; Stasimioti and Sosoni, 2021). Studies have also addressed the issue of machine translation acceptability (see Castilho, 2016; Castilho and O'Brien, 2018; Rivera-Trigueros, 2021; Taivalkoski-Shilov et al., 2022). However, the attitudes and perceptions of translation students, novice translators, professional translators and posteditors have been mainly taken into the focus, possibly due to a somewhat easier access to respondents and more convenient research design (see Moorkens and O'Brien, 2015; Rossi and Chevrot, 2019; Ferreira et al., 2021). The acceptability of machine-translated content by non-professional users has not been extensively studied. The ordinary users' perspective is important because of the variety of purposes for which they take machine translation for granted and use it daily (Kasperė and Motiejūnienė, 2021).

The study^1^ seeks to investigate the acceptability of raw machine translation texts in Lithuanian, a low-resource language. In this paper, we report the results of an eye tracking experiment with professional translators and non-professional users of machine translation with the focus on acceptability. The inter-group and intra-group comparisons of raw machine-translated text acceptability are made. The research questions are as follows: do non-professional users process raw machine translation output in the same way as professional users? Is there a difference in the processing of raw machine-translated output among non-professional users with different levels of acceptability of machine-translated text? Is there a difference between professional and non-professional users' comprehension of the raw machine-translated output?

## 2. Literature overview

Machine translation acceptability is a multilayered concept. Criteria of usability, satisfaction and quality have been indicated to be the components of acceptability. Castilho and O'Brien (2018) define acceptability as machine translation output quality in terms of correctness, cohesion and coherence from the reader's perspective. Even if the text contains errors, it does not mean that it is considered unacceptable. If the needs of the readers are satisfied, the text has served its mission (Castilho and O'Brien, 2016, 2018). In order to measure acceptability, Castilho (2016) defines the three criteria. Usability is related to efficiency and effectiveness of the text and may be measured by exerted cognitive effort; satisfaction, which is understood as a user's positive attitude toward the translated text, may be measured through web surveys, post-task satisfaction questionnaires or moderators' ratings; and quality is defined by fluency, adequacy, syntax and grammar, and style in translated content or as text easeability, readability, etc. (Castilho, 2016). For the purposes of this research, acceptability is understood as a notion combining satisfaction, usability and quality as assumed by the ordinary readers of the text who have no linguistic background or related, e.g., translator, training.

Research employing eye tracking methodology is common in Translation Studies (Carl et al., 2011; Castilho, 2016; Daems et al., 2017; Moorkens, 2018; Vardaro et al., 2019; Ferreira et al., 2021; Stasimioti and Sosoni, 2021). Among the existing body of scientific literature on the acceptability criteria of machine translation, of particular mention are those published papers that employ eye tracking experiments. Since acceptability is a vague notion representing quite subjective understanding and judgement, eye tracking studies present relevant insights into the readers' cognitive processing of the (machine-translated) text they are reading. The research reveals that the required cognitive load is generally to a greater or lesser extent higher in cases where machine translation is provided in comparison with human-translated or post-edited text.

Jakobsen and Jensen (2008) report the results of a translation process study, focusing on the differences between the reading of a text with the aim of understanding its meaning and reading the same text (or a very similar text) with the expectation of having to translate it next. The authors recorded eye movements of six translation students and six professional translators who were asked to perform four tasks at the speed at which they normally work, namely read a text for comprehension, read a text in preparation for translating it later on, read a text while performing its oral translation and read a text while typing a written translation. The researchers compared task duration, total number of fixations, total gaze time and average duration of individual fixations for each task and found out that the purpose of reading had a clear effect on eye movements and gaze duration. Overall, the increases in the number of fixations from the first to the last task of the experiment were statistically significant (Jakobsen and Jensen, 2008).

In a study by Guerberof Arenas et al. (2021), researching the effect of different translation modalities on users through an eye tracking experiment, 79 end users' (Japanese, German, Spanish, English) experiences with published translated, machine-translated and published English versions were compared. The authors focused on the number of successful tasks performed by end users, the time necessary for performing successful tasks in different translation modalities, the satisfaction level of end users in relation to different translation modalities and the amount of cognitive effort necessary for carrying out tasks in different translation modalities (Guerberof Arenas et al., 2021). They measured usability, i.e., effectiveness (by asking participants to perform some tasks), efficiency (by measuring the time to complete the tasks and by measuring cognitive effort using an eye tracker) and satisfaction. The authors came to the conclusion that the effectiveness variable was not found to be significantly different when the subjects read the published translated version, a machine-translated version and the published English version of the text although efficiency and satisfaction were significantly different, especially for less experienced participants. The results of the eye tracking experiment revealed that end users' cognitive load was higher for machine-translated and human translated versions than for the English original. The findings also indicated that the language and the translation modality played a significant role in the usability, regardless of whether end users finished the given tasks and even if they were unaware that MT was used (Guerberof Arenas et al., 2021).

In a study by Hu et al. (2020), an eye tracking experiment involving 66 Chinese participants with low proficiency in English who also had to fill in questionnaires on comprehension testing and attitudes showed that the quality of raw machine-translated output was considered somewhat lower, but almost as good as that of a post-edited machine-translated output, although the research design involved non-professional post-editing of machine-translated text.

Some earlier user-centered studies where raw machine translation was analyzed *via* eye tracking, screen recording experiments and post-task questionnaires determined a lower usability of machine-translated instructions in comparison with post-edited output (Castilho et al., 2014; Doherty and O'Brien, 2014; Doherty, 2016).

In a study of non-professional users where acceptability of a machine-translated text from English into Lithuanian was tested, an eye tracking experiment revealed that the cognitive processing was greater, i.e., required a longer gaze time and fixation count, on machine translation errors in comparison with correct segments of text (Kasperavičienė et al., 2020). The machine-translated segments with errors required more attention and cognitive effort from the readers, but the results regarding overall acceptability of the raw machine-translated text obtained *via* a post-task survey did not correlate with the readers' gaze time spent on segments with errors.

Literary texts have also received some attention with regard to the differences between human and machine translations from English into Dutch as perceived by end users. Colman et al. (2021) employed eye tracking to analyze end users' reading process and determine the extent to which machine translation impacts the reading process. An increased number of eye fixations and increased gaze duration while reading machine translation segments was found in comparison with human translation (Colman et al., 2021).

Although scarce, there is some research, based on research designs employing methodologies other than eye tracking, determining how the acceptability of machine-translated texts in various languages is perceived by non-professionals or low proficiency future professionals. The broad public uses machine translation for many reasons and purposes and they may not fully understand or consider how machine translation really works and what quality it generates. In a study of 400 surveyed participants, acceptability of the text that had been machine translated from English to Lithuanian was found to be affected by such factors as age and education. The less educated and senior participants were more prone to consider machine translation reliable and satisfactory (Kasperė et al., 2021).

In a study by Rossetti et al. (2020), 61 participants were surveyed in order to get insight into the “impact of machine translation and postediting awareness” on comprehension and trust. The participants were asked to read and evaluate crisis messages in English and Italian using ratings and open-ended questions on comprehensibility and trust. The authors found insignificant differences in the end users' comprehension and trust between raw machine-translated and post-edited text (Rossetti et al., 2020). However, users with low proficiency of English were more positive toward raw machine-translated text in terms of its comprehension and trust (Rossetti et al., 2020).

In another study with translation agencies, professional translators and clients/users of professional translation, the level of user awareness of machine translation was studied through surveys (García, 2010). Acceptability and evaluation of machine translation from Chinese into English was at the focus. The researcher found out that <5% of professional translators considered the quality of machine translation very high. The translation agencies expressed a very similar view on machine translation to that of the translators. The clients/users of professional translations (about 30%) who were aware of and requested machine translation had an intermediate or positive assessment of the quality of machine translation (García, 2010).

As the amount of content to be translated is growing, there is a demand to cut the cost of translation orders, which leads to a growing need for research and testing how translators work with machine translation (Moorkens and O'Brien, 2015) and the newly-arising need to learn how the end users are aware of, perceive, use and accept machine-translated content.

## 3. Materials and methods

Machine translation quality overall can be assessed in various ways: by applying automatic quality estimation metrics, by carrying out an error analysis by professionals/experts, employing cognitive experimental methods with human experts or professionals or semi-experts or non-experts, determining acceptability of the output of non-experts/non-professionals/amateur users, *via* qualitative methods, etc. Recently, cognitive experimental methods for machine translation quality assessment have been increasingly employed, e.g., eye tracking, key logging, screen recording, post-performance (retrospective) interviews, think-aloud protocols, etc. In an eye tracking experiment, fixation count and time, gaze time, saccades, pupil dilation, and other variables can be measured, although researchers have determined that, for example, pupil dilation may not adequately reflect cognitive effort involved or provide valid and reliable data. To test the validity of the data, cognitive translation researchers have employed complementary methods, including other experimental methods, interviews or surveys. Translation research studies employing eye tracking have mostly relied on post-performance or retrospective interviews/surveys, and the number of subjects involved in an eye tracking experiment for translation research varies between 2 and 84 (per language). The most common eye movement measures taken into account and described in translation research are fixation time and fixation count (Kasperė and Motiejūnienė, 2021).

### 3.1. Experiment

For the current study, we used an eye tracking experiment along with a questionnaire in order to ensure the validity of results obtained. Before the experiment, a larger-scale population survey was conducted to find out the purposes, typical circumstances of machine translation use and systems employed non-professional users (Kasperė et al., 2021). In the survey, the respondents were asked to indicate a machine translation tool that they used most often. The reported results of the survey revealed that the absolute majority of the respondents indicated that they used Google Translate as the tool for machine translation (Kasperė et al., 2021). We, therefore, also employed it for the machine translation of the text in the research design of this particular study. Google Translate has over 500 million users per month and over 140 billion words are translated per day (Schuster et al., 2016; Hu et al., 2020). The text chosen for a reading task in the experiment was a recipe of a dish. The motivation behind selecting the text of a recipe for this experiment lies in the findings of the above-mentioned study where the respondents indicated various reasons for using machine translation in their everyday activities, one of the most common being household purposes (Kasperė et al., 2021). The text of a recipe, originally in English, was machine translated using Google Translate to Lithuanian. The translated excerpt given to the subjects as a reading task contained 371 words and was arranged on three slides 13–15 lines each.

In the machine-translated excerpt, we selected areas of interest with errors and areas of interest without errors. According to scientific literature, the perceptual span in western languages is about 13–15 characters to the right of the center of vision, and 3–4 to the left (McConkie and Rayner, 1975; Rayner, 1998). Therefore, all our selected areas of interest (both with and without errors) included 18–20 characters. In the raw translated text prepared for the experiment, 12 distinct errors were selected as areas of interest. Another 12 areas of interest without errors were selected as control. To identify the errors, we used the Multidimensional Quality Metrics, which is a typology of errors developed for assessment of the quality of human translated, machine translated and post-edited texts. This system covers more than 100 error types and can be adapted to all languages (Lommel et al., 2014). Within this classification, the following main types of errors are as indicated: terminology; accuracy (for example, addition, mistranslation, omission, untranslated text, etc.); linguistic conventions (also called fluency in the previous versions of the taxonomy, related to errors in grammar, punctuation, spelling, unintelligible text, etc.), design and markup (errors related to visual presentation of a translated text, such as text formatting, layout); locale conventions (errors related to locale-specific content); style (errors related to inappropriate organizational or language style); and audience appropriateness (for example, errors related to culture-specific reference) (MQM Commitee, 2022). The 12 identified errors fell into two 2 different categories of errors, namely accuracy and linguistic conventions. Accuracy errors were those of mistranslation, untranslated text, omission, and addition. Errors that fell within the linguistic conventions category were those of an incorrect word form (ending) resulting in inappropriate agreement between the words in a phrase.

Eye tracking was performed using a commercial non-invasive eye tracking device SensoMotoric Instruments GmbH Scientific RED-B.6-1524-6150133939 and SMI BeGaze 3.7.42 software for data analysis. For each area of interest (AOI), several eye movement measures were taken into consideration: fixation time (total time of fixations that happened in the AOI), dwell time (total time of fixations and saccades that happened in the AOI), and glance count (the number of times when the gaze entered the AOI).

### 3.2. Research participants

In total, there were 30 subjects in the experiment: 11 professional translators, language editors and revisers and 19 non-professional users of machine translation, who were of different educational backgrounds, age, occupation. All subjects were native speakers of Lithuanian. Among the non-professional users, 13 had a university degree and 6 had secondary education. The subjects gave consent to participate in the experiment on a voluntary basis. They were informed that the text they were reading was a machine translation. The subjects were also told that they would have to answer questions about the text afterwards filling in a post-task questionnaire. There were 4 reading comprehension questions, all related to the errors in the text, including 2 true/false questions and 2 open questions. The post-task questionnaire also had 9 statements, 3 per each component of acceptability (i.e., quality, usability and satisfaction). The statements could be assessed by the subjects on a 5-point Likert scale, where 1-completely disagree, 2-somewhat disagree, 3-neither agree nor disagree, 4-somewhat agree, and 5-completely agree. In total, in this part of the questionnaire, the subjects of the experiment could accumulate a maximum of 45 points: 15 for quality, 15 for usability and 15 for satisfaction. The questions and the statements provided to the subjects in a post-task questionnaire were presented in their native, i.e., Lithuanian, language.

### 3.3. Data analysis

IBM SPSS Statistics 27 was used for descriptive and relationship analysis. Descriptive statistics were calculated for quantitative nominal and ordinal data. The relationships between data were investigated using column plots and box plots. Although the convenience sample was used, limiting the usefulness of hypothesis testing, several non-parametric tests (one-sample Kolmogorov-Smirnov test, independent-samples Mann-Whitney *U*-test, independent-samples Moses test of extreme reaction) with a significance level of 0.05 were used to explore what hypotheses would be more promising for further research.

## 4. Results

**Figure 1:**
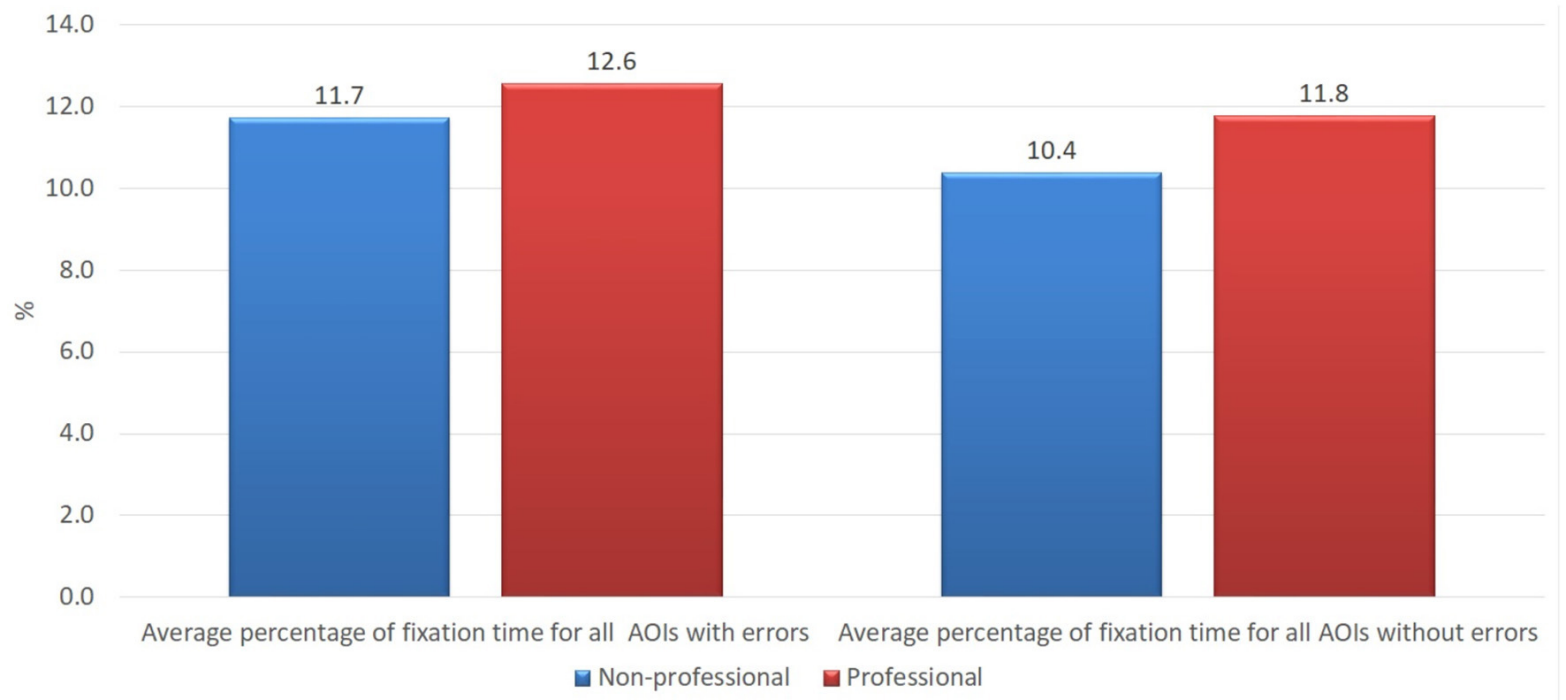
Average percentage of fixation time on all areas of interest with errors and without errors in the groups of professional and non-professional users of machine translation.

The findings of our study demonstrate that the average fixation time on the areas of interest with errors of both groups of the subjects was longer than on the areas of interest without errors, which confirms findings of other studies that errors attract more readers' attention and require more cognitive effort than correct text (see Figure 1). The average fixation time on the areas of interest with errors (in percentage from total time of the trial) was 12.6 vs. 11.7% for professional and non-professional users of machine translation, respectively. On the other hand, professionals also demonstrated a longer average fixation time on areas of interest without errors than non-professionals, i.e., 11.8 vs. 10.4%. The longer average fixation time on both types of areas of interest within the professionals' cohort might be interpreted that professional translators and language specialists who work with texts on a daily basis have different skills and a more pronounced critical look at any text. Such a hypothesis would still have to be tested on a broader scale experiment.

Independent-samples Mann-Whitney *U-*test would indicate that the hypothesis that the fixation time of professionals and non-professionals for AOIs with errors has the same distribution (more precisely, the hypothesis that the probability of fixation time being higher for a random professional than for random non-professional is 0.5) could not be rejected (*p* = 0.792). Still, the independent-samples Moses test of extreme reaction suggests that, while hypothesis about the distributions having the same range could not be rejected, the value of *p* is much closer to the level of significance (*p* = 0.079). Similar (although weaker) relationship holds for AOIs without errors (*p* = 0.670 and *p* = 0.180).

**Figure 2:**
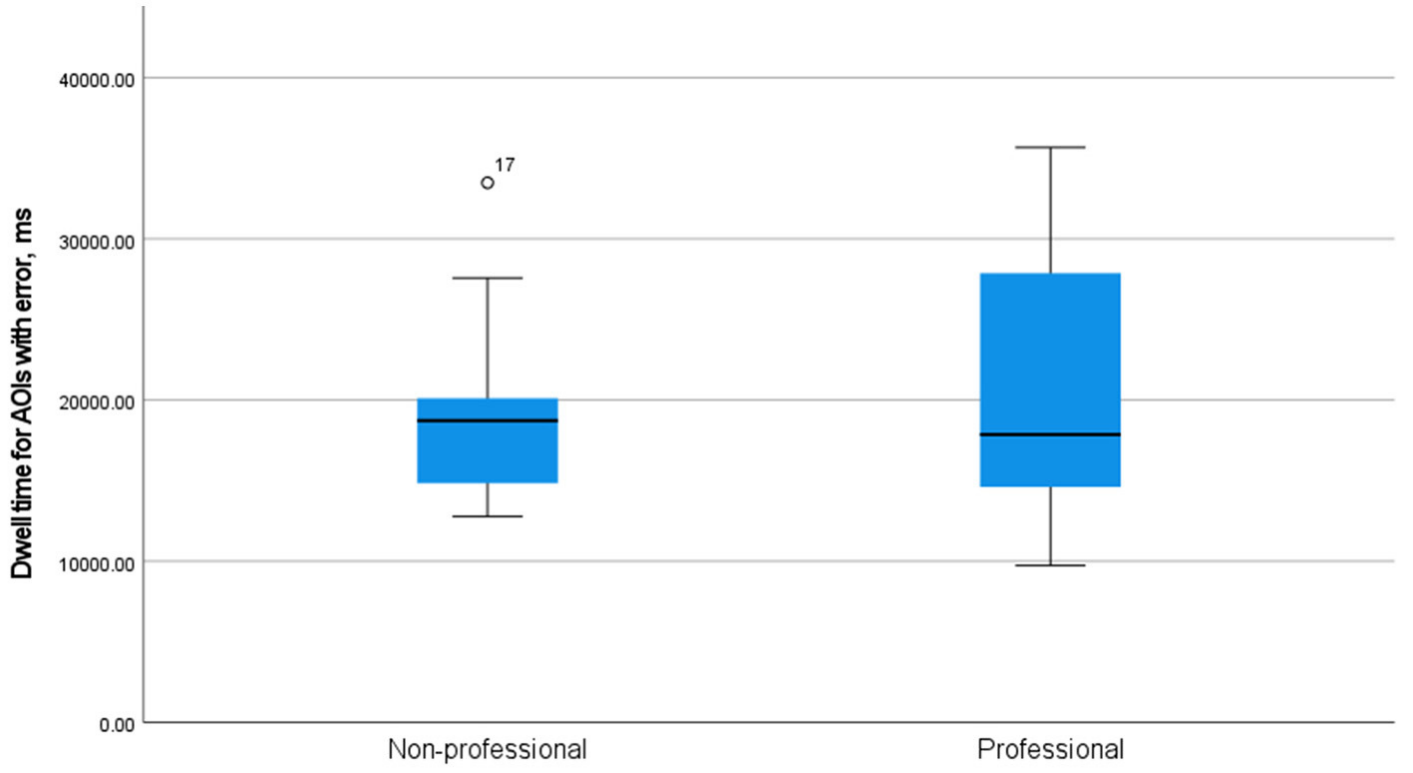
A simple boxplot of dwell time on AOIs with errors in the groups of professional and non-professional users of machine translation.

As Figure 2 shows, while the median of dwell time for AOIs with errors was very similar for professionals (17,851 ms) and non-professionals (18,722 ms), the spread of it was clearly different. That might be assumed to be rather surprising, for, intuitively, one might suppose that professionals are going to be more like each other than non-professionals.

Different eye movement measures, including the fixation time, were also compared in the groups of the subjects who scored high and low in the post-task survey for the questions demonstrating quality and usability of the text and the users' satisfaction with the text.

**Figure 3:**
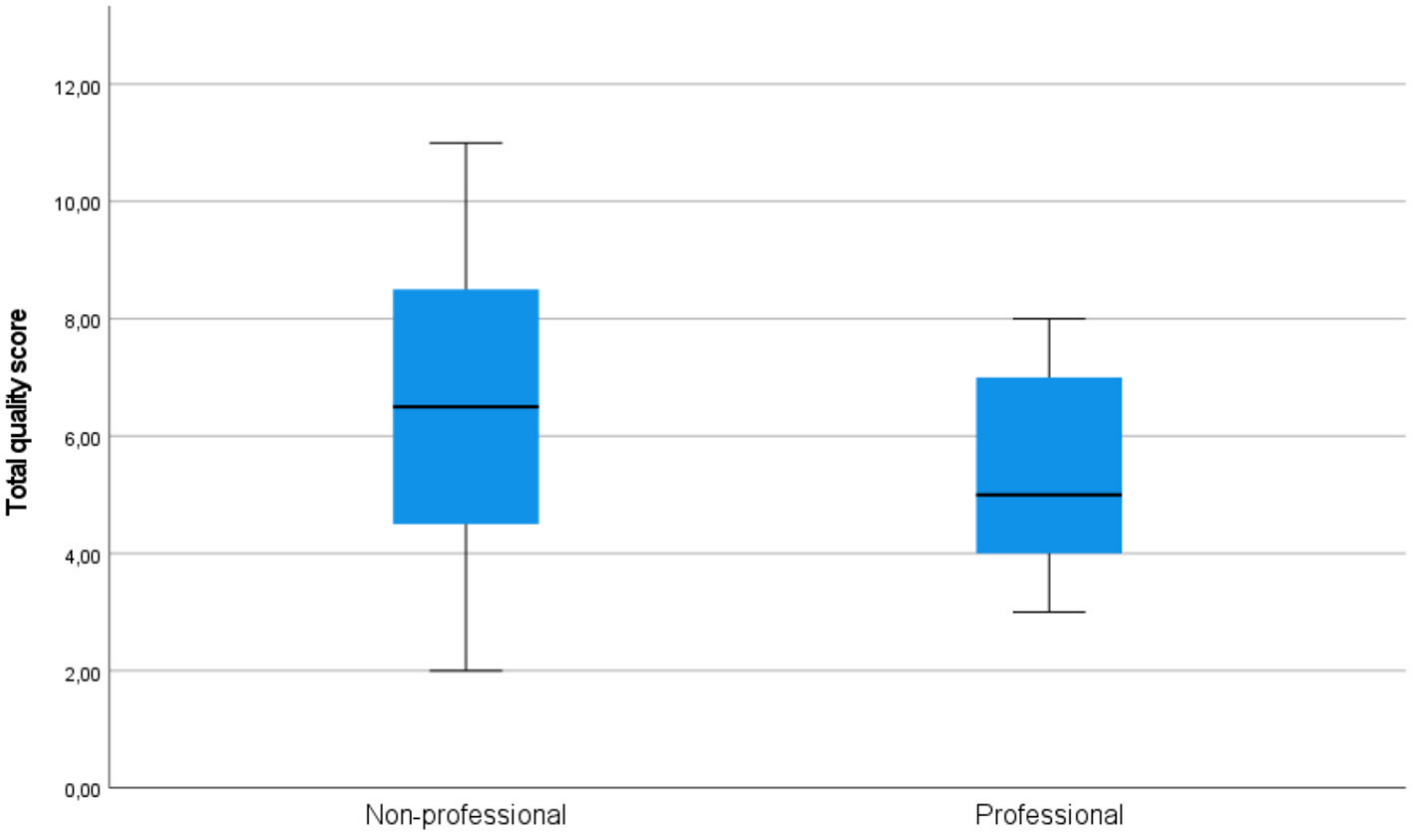
Total quality scores in the groups of professional and non-professional users of machine translation.

**Figure 4:**
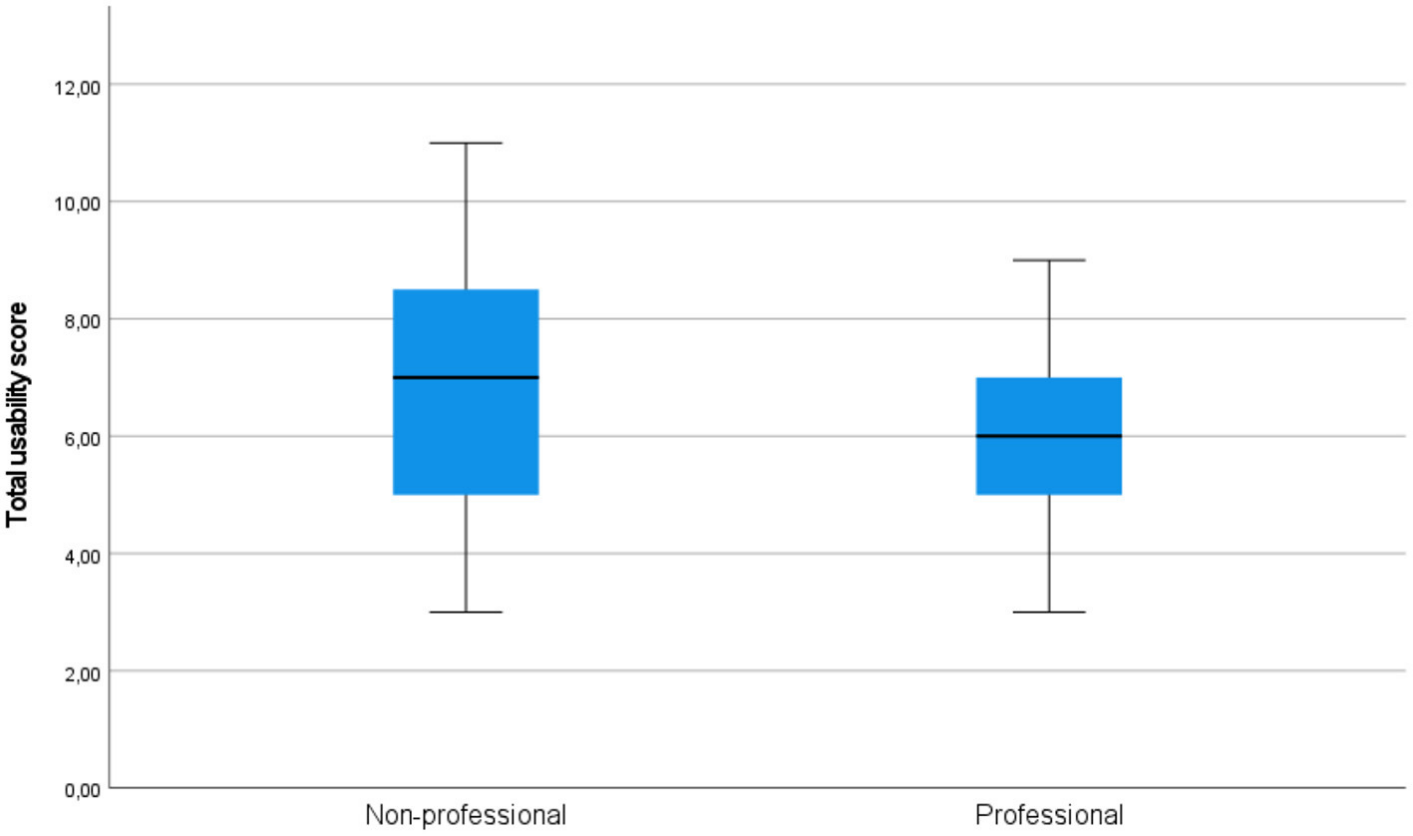
Total usability scores in the groups of professional and non-professional users of machine translation.

**Figure 5:**
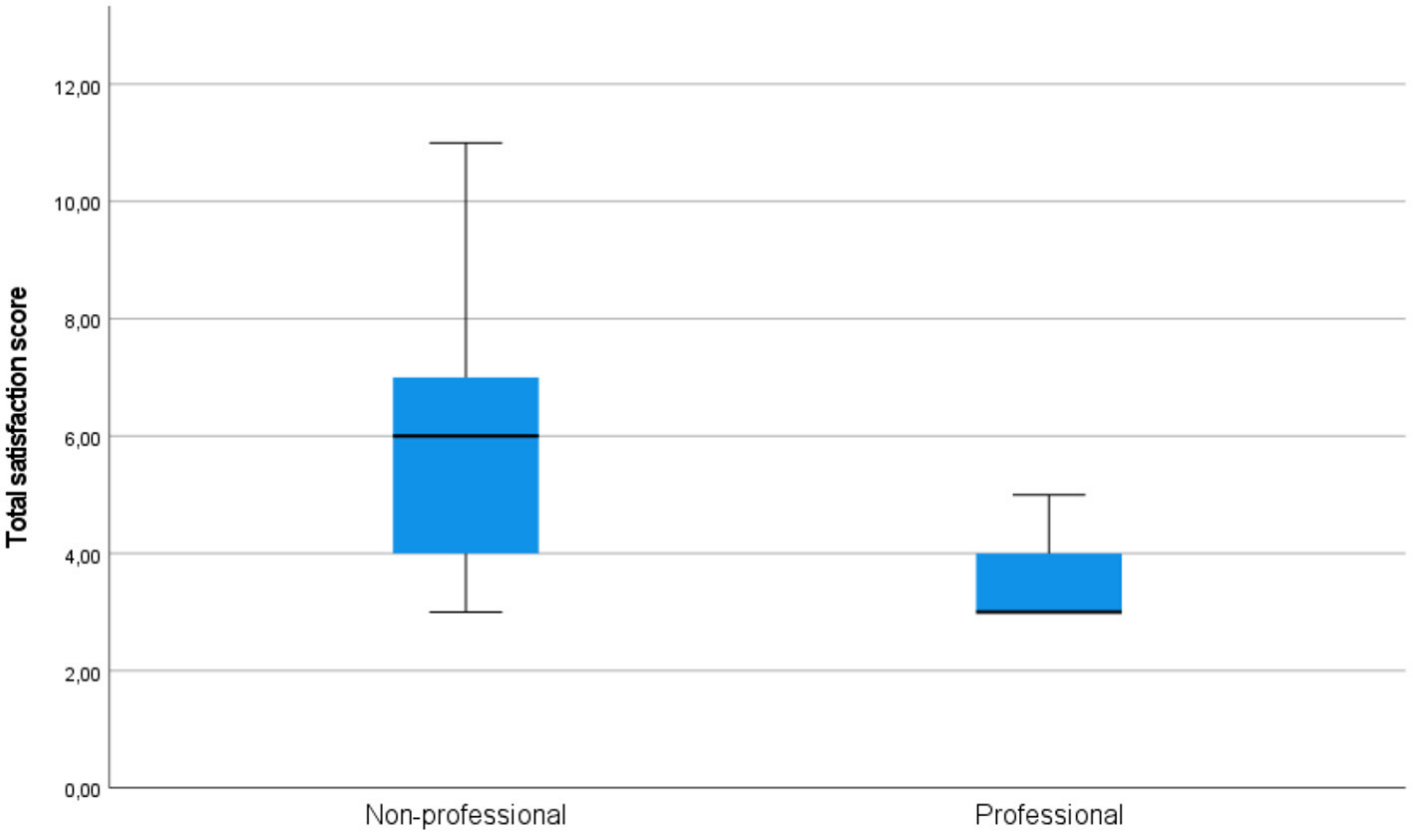
Total satisfaction scores in the groups of professional and non-professional users of machine translation.

On all components of acceptability (see Figure 3 for quality, Figure 4 for usability, and Figure 5 for satisfaction), non-professional users scored higher than professionals. The average total quality scores were 6.2632 for non-professionals and 5.3636 for professionals (median 6 vs. 5, respectively). The average total usability scores were 6.6842, i.e., slightly better, for non-professionals compared with professionals, i.e., 6.0000 (median 7 vs. 6, respectively). In terms of the average total satisfaction scores, the non-professionals' scores were much more increased compared with professionals, i.e., 5.7895 vs. 3.5455 (median 6 vs. 3), respectively. This suggests that non-professional users were more positive toward the machine-translated text than professional users, perhaps because professional users are more aware of features of good translation and are able to notice when they are not present.

Independent-samples Mann-Whitney *U*-test would also indicate that the hypothesis that the total satisfaction score of professionals and non-professionals has the same distribution (more precisely, the hypothesis that the probability of this score being higher for a random professional than for a random non-professional is 0.5) can be rejected (*p* < 0.001). On the other hand, the same test does not suggest rejecting the hypotheses that the total usability score and the total quality score of professionals and non-professionals have the same distributions (*p* = 0.427 and *p* = 0.381).

One-sample Kolmogorov-Smirnov test suggests that the hypotheses of total quality score, total usability score and total satisfaction score having normal distribution could be rejected (*p* = 0.020, *p* = 0.017, *p* < 0.001), while the hypothesis that their sum has a normal distribution could not be rejected (*p* = 0.200).

**Figure 6:**
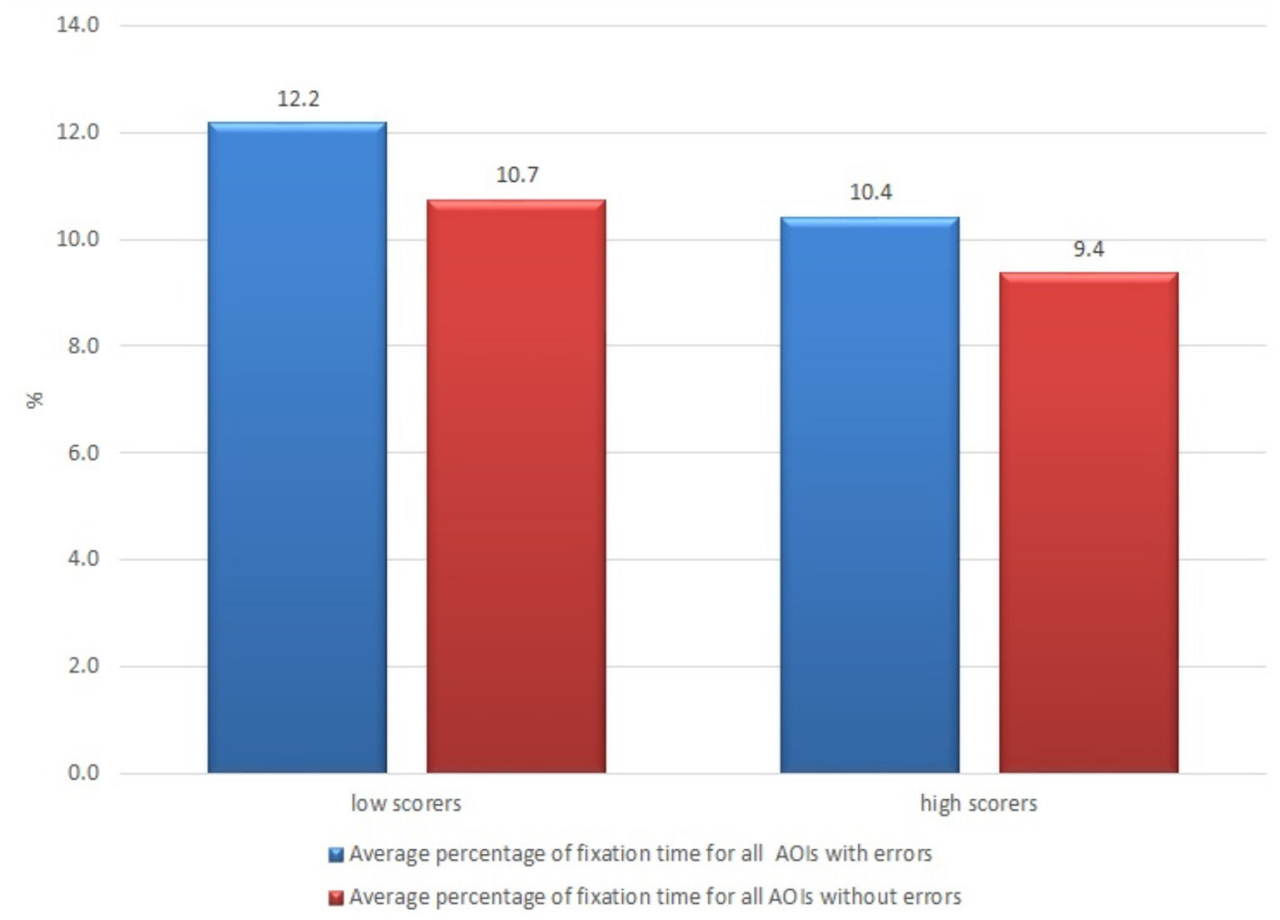
Average percentage of fixation time of non-professional users who rated quality of the raw machine-translated text higher and lower than the average.

The subjects from the group of non-professional users who thought that the quality was low (having scores lower than average; there were 16 such subjects out of 21) demonstrated a longer average fixation time both for AOIs with errors and without errors (12.2 vs. 10.7%, respectively) than those subjects who thought that the quality was high (10.4 vs. 9.4%, respectively) (see Figure 6).

**Figure 7:**
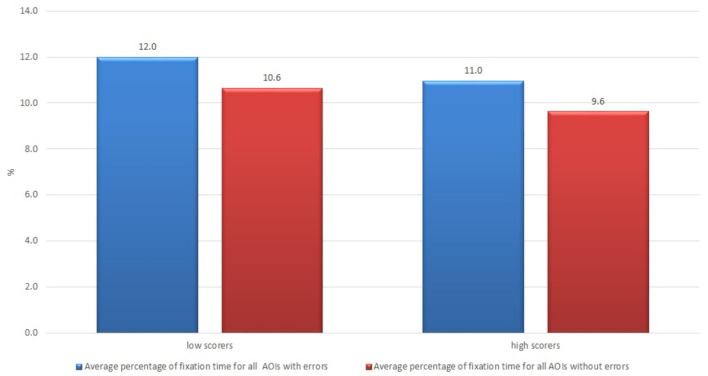
Average percentage of fixation time of non-professional users who rated usability of the raw machine-translated text high and low.

The same pattern was observed for the usability and satisfaction components. The subjects who thought that the text was barely usable (having scores lower than average; there were 16 such subjects out of 21) showed a longer fixation time result that those who thought that the text was usable (12.0 vs. 11.0% and 10.6 vs. 9.6%, respectively) (see Figure 7).

**Figure 8:**
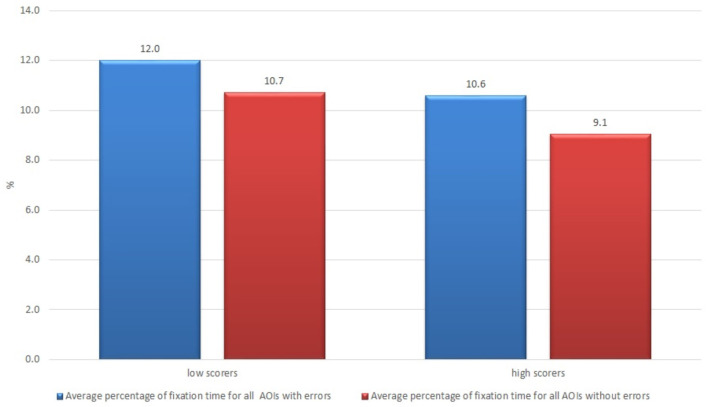
Average percentage of fixation time of non-professional users who were more and less satisfied with the raw machine-translated text.

The non-professional users who were less satisfied with the text (value lower than average; there were 17 such subjects out of 21) demonstrated a longer average fixation time result in comparison with those who were more satisfied with the text (12.0 vs. 10.6%, respectively) (see Figure 8).

**Figure 9:**
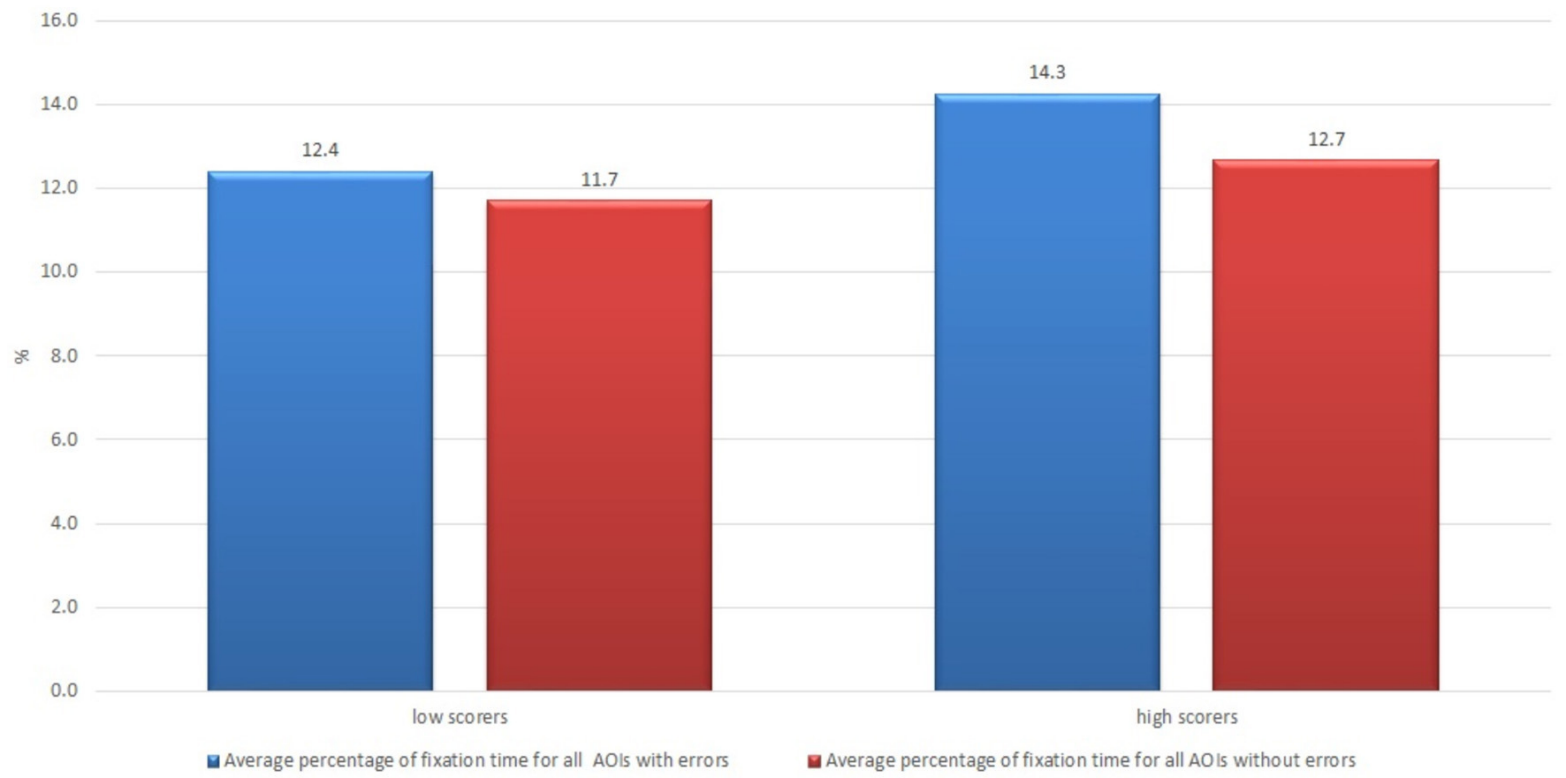
Average percentage of fixation time of professional translators who scored low and high regarding the usability with the raw machine-translated text in the post-task questionnaire.

All professional translators, language editors and revisers who read the raw machine-translated text provided to them in the experiment thought that the text quality was low, and they scored low on the questions of satisfaction in the post-task questionnaire on acceptability components. Only in terms of usability, the subjects of the professional translators' group were divided into those who thought that the text was usable to some extent (usability higher than average) and those who thought that the text was not usable. The results for average percentage of fixation time of the two groups of professional translators - low scorers and high scorers for usability statements—are shown in Figure 9. The subjects in the group of low scorers for the usability statements demonstrated a shorter average fixation time compared with those who scored higher, 12.4 vs. 14.3% for AOIs with errors and 11.7 vs. 12.7% for AOIs without errors, which also raises questions for further research, discussion and implications.

**Figure 10:**
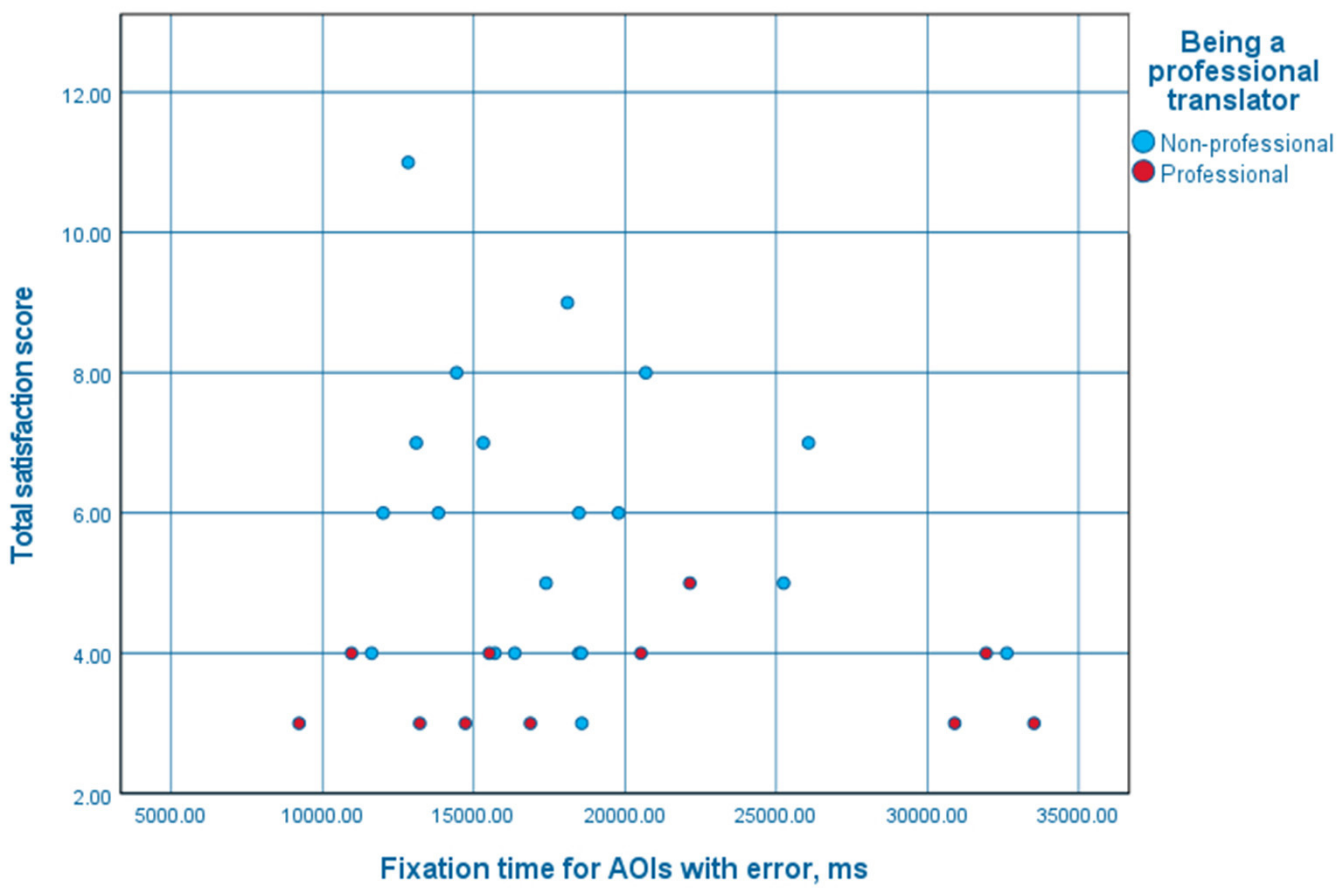
A scatter plot of fixation times for AOIs with errors and total satisfaction scores.

As Figure 10 shows, total satisfaction scores for non-professionals who looked at AOIs with errors for a shorter period of time than average varied greatly. The higher limit of those scores decreased for non-professionals who looked at such AOIs longer, while the lower limit tended to stay the same. On the other hand, the satisfaction scores for the professionals tended to stay the same, as for non-professionals who paid more attention to the AOIs with errors.

However, the independent-samples Mann-Whitney *U*-test would indicate that the hypothesis that the total satisfaction score of professionals and non-professionals has the same distribution (that the probability of this score being higher for a random professional than for a random non-professional is 0.5) cannot be rejected (*p* = 0.157).

**Figure 11:**
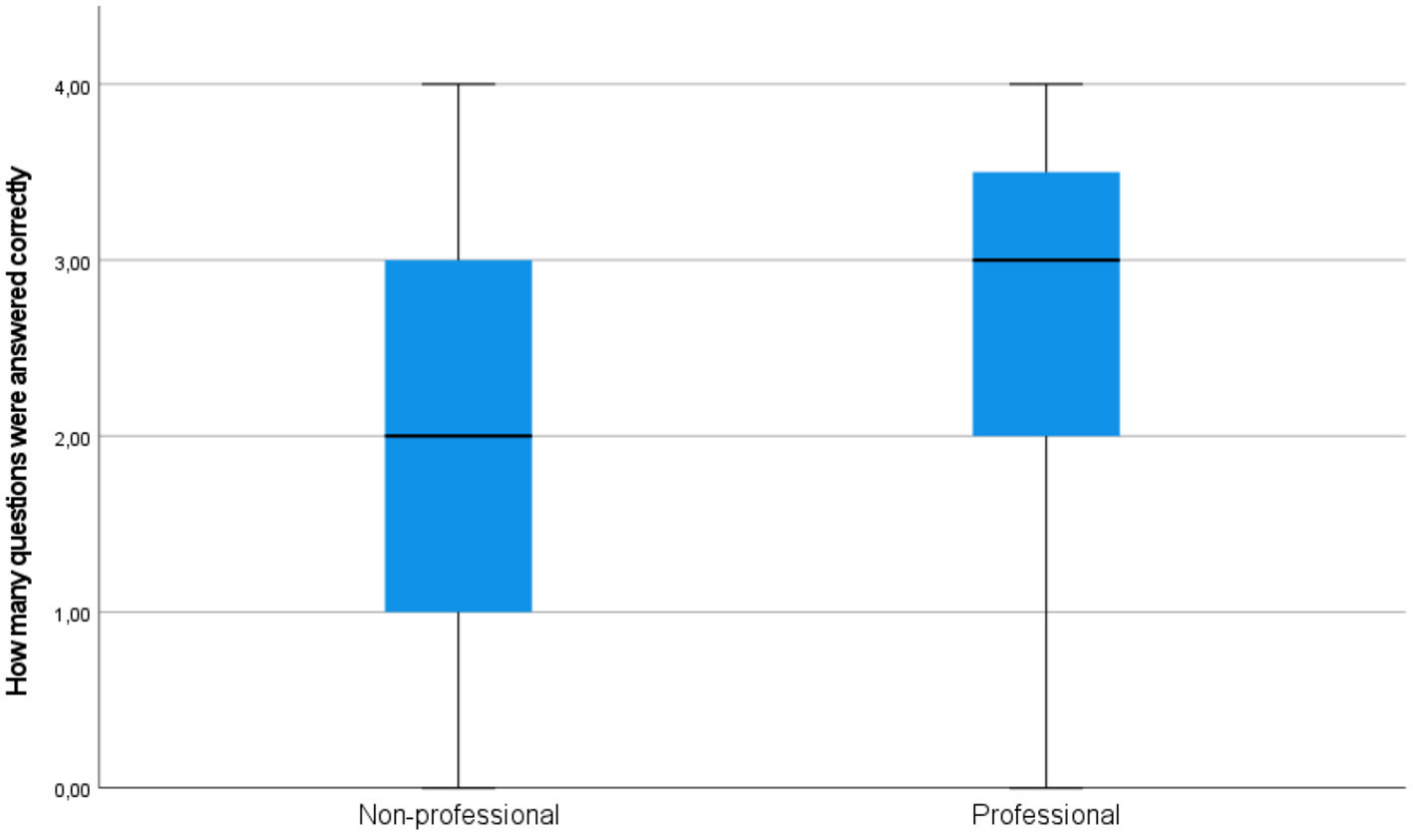
A simple box plot of text comprehension results in the groups of professionals and non-professional users.

Besides, the subjects' text comprehension was measured *via* a post-task reading comprehension questionnaire, consisting of 4 questions, i.e., 2 true/false questions and 2 open questions. Figure 11 demonstrates how the subjects scored in both groups. The professionals scored better in text comprehension compared with non-professional users (median 2 vs. 3, respectively) (see Figure 11).

**Figure 12:**
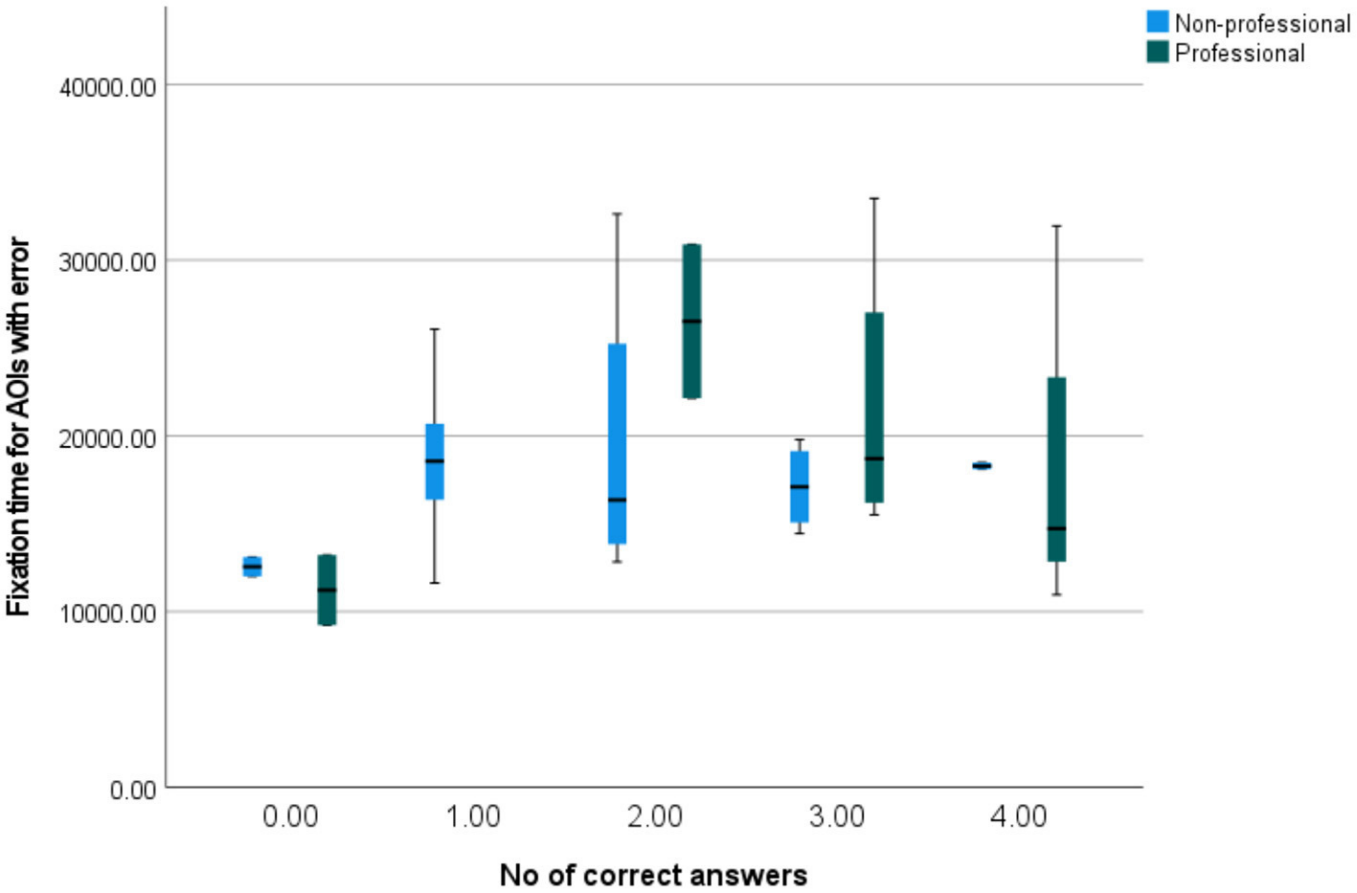
A simple box plot of fixation times for AOIs with errors by the number of correctly answered questions.

Figure 12 shows how fixation times for AOIs with errors correlate with the number of correctly answered questions. It may be seen that the pattern differs between professionals and non-professionals, with professionals having higher spread for more correct answers and non-professionals having higher spread for average number of correct answers. It is also interesting that the median dwell time was mostly the same for non-professionals giving different numbers of correct answers, while the median dwell times for professionals giving the highest and the lowest numbers of correct answers are lower than for professionals who gave a medium number of correct answers. Furthermore, both professionals and non-professionals who gave no correct answers (there were two such professionals and two such non-professionals) had low dwell times (with the maximum lower than the medians of every other group).

**Figure 13:**
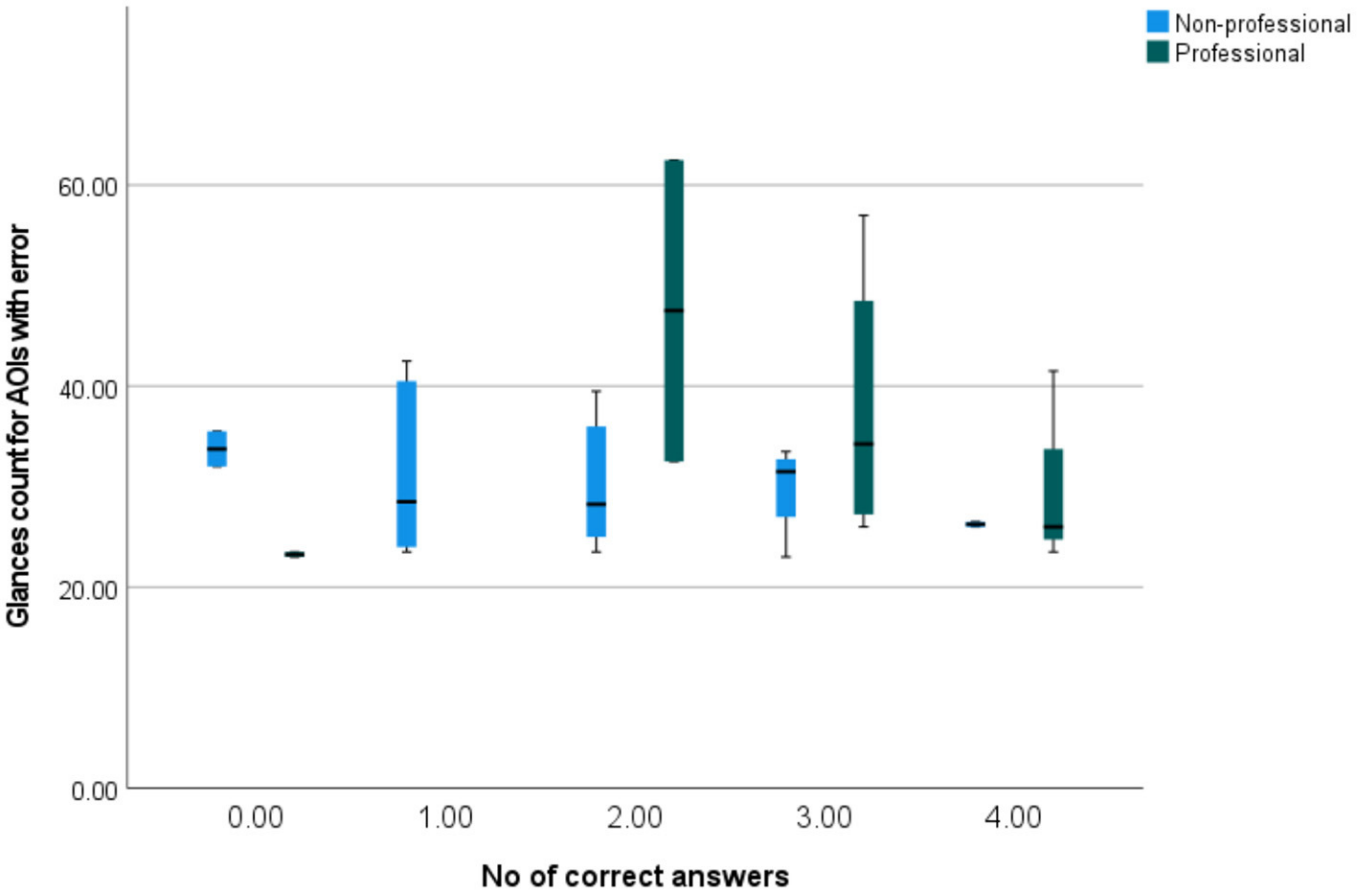
A simple box plot of glances counts for AOIs with errors by the number of correctly answered questions.

Figure 13 shows how glance counts for AOIs with errors correlate with the number of correctly answered questions. The differences between professionals and non-professionals may be observed, with professionals having higher spread and non-professionals having lower spread for the higher number of correct answers. Professionals tended to reach higher glance counts (for each number of correct answers, professionals tended to have a higher median glance count, with the exception of the group of no correct answers, which might have been an outlier). Furthermore, non-professionals who gave no correct answers had high glances counts (with median higher than the medians of every other group of non-professionals). As they also had low dwell times, this might indicate that the respondents who gave no correct answers were relatively inattentive.

**Figure 14:**
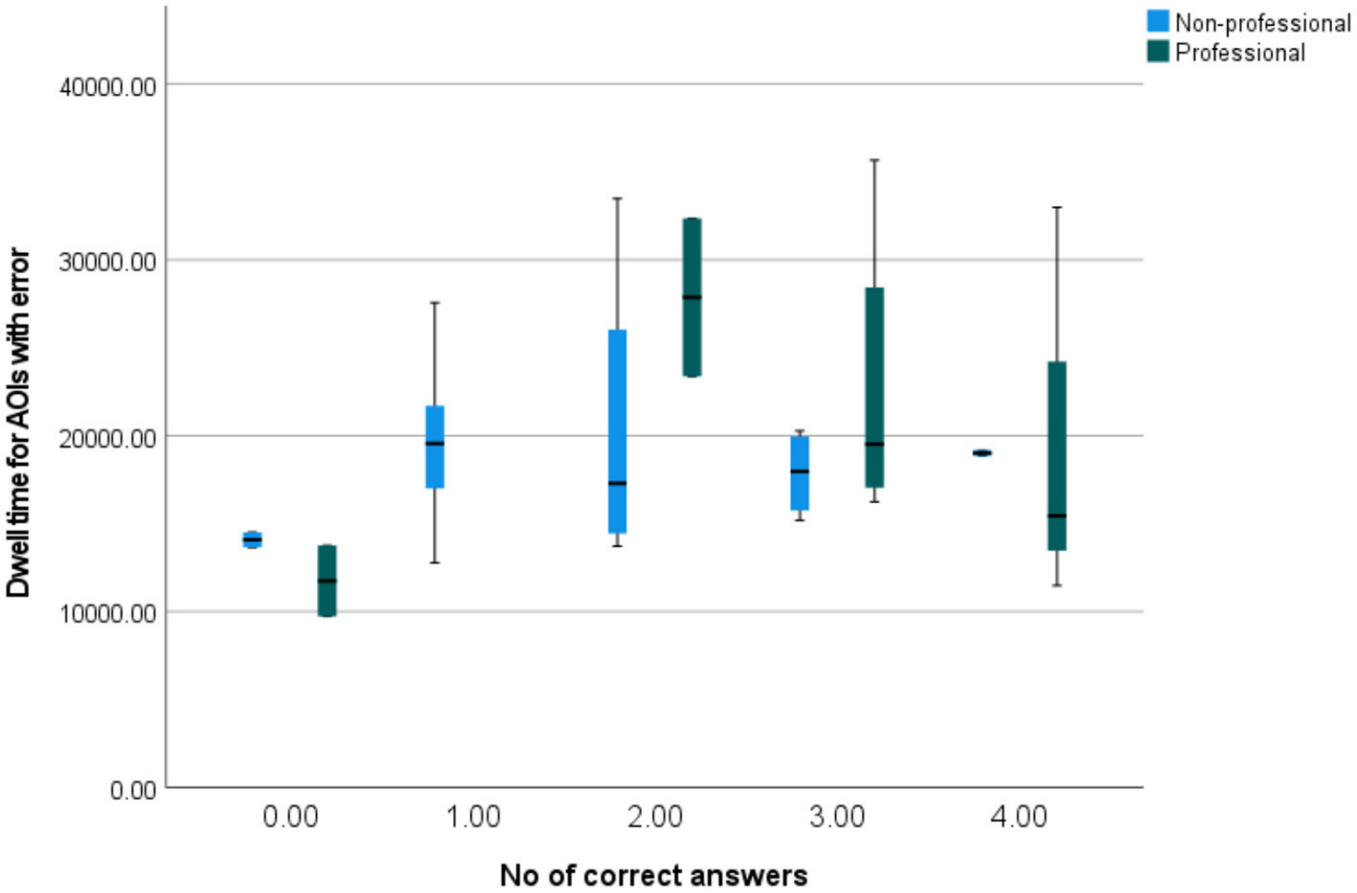
A simple box plot of dwell times for AOIs with errors by the number of correctly answered questions.

Figure 14 shows how dwell times for AOIs with errors correlate with the number of correctly answered questions. The pattern again differs between professionals and non-professionals, with professionals having a higher spread for more correct answers and non-professionals having a higher spread for the average number of correct answers. Furthermore, both professionals and non-professionals who gave no correct answers had low fixation times (with the maximum lower than the medians of every other group), which may imply that less attention and effort while reading results in lower comprehension. Of course, such a finding needs to be tested and proven in a better targeted study, as in this particular case there might have been other factors like the text type, topic, tiredness, general absence of interest, etc. that influenced the results.

## 5. Discussion

Previous studies focusing solely on machine translation acceptability are few. Even fewer studies apply eye tracking to test machine translation acceptability. They mainly focus on the experiments with professional translators and/or translation students. To the best of our knowledge, there are no reported studies where machine translation acceptability by non-professional users was tested *via* an eye tracking experiment. No such research testing acceptability of machine-translated text into Lithuanian has been conducted so far. Lithuanian, like many other smaller languages, is considered underresourced. It is also a morphologically rich synthetic language. Consequently, machine translation quality is less adequate than in other languages where investment into data acquisition and machine translation development is more substantial. Therefore, the views of Lithuanian language speakers, or smaller language speakers overall, toward machine translation might be diverse and involve many more risks or unexpected threats, if the output is used without critical awareness and judgment. For these reasons, comparisons between our results and previous research are only partial or indirect.

This study revolved around three research questions. The first question was related to comparison between professional and non-professional users' processing of raw machine translation output. The most obvious finding to emerge from this study is that there is a difference in the machine translation output cognitive processing and acceptability between professional and non-professional users. In comparison with non-professional users, professional users of machine translation, i.e., translators and language editors, spend more time overall reading the machine-translated texts, most probably because of their deeper critical awareness as well as proficient attitude toward the text. They also demonstrate a longer average fixation time and a greater average glance count on the machine translation errors. In terms of acceptability overall, professional users critically assess machine translation on all components of acceptability. This might possibly be explained by an assumption that professionals have less tolerance toward insufficient quality of machine translation, know how to prepare texts for publishable quality and see mistakes, inaccuracies and style issues in a text almost instantaneously. On the other hand, even if the text contains errors, it might still be usable.

The results obtained in this study seem to be to some extent consistent with the findings obtained in previous studies. García (2010) who investigated the level of user awareness of machine translation among professional translators and clients or users of translation found out that only a small proportion of professionals considered the quality of machine translation very high, which is not surprising since at the time machine translation had lower quality than the neural machine translation now. However, in the same study, the clients/users of translations demonstrated more positive assessment of the quality of machine translation compared to that of professional users (García, 2010). Our findings are also in line with the implications revealed by Vieira (2020) who concluded that there is a clear divide between the perceptions of professionals and non-professionals toward machine translation and its capabilities. In his study, Vieira acknowledged that the public coverage of machine translation veers more toward positive attitudes rather than negative. In our study, non-professional users—end-users with no linguistic background—also had more positive attitudes toward machine translation quality, usability and satisfaction compared with the professional translators' attitudes toward the text. However, in principle, our results may also be indirectly considered to be in agreement with those obtained in a study by Hu et al. (2020) where subjects with low proficiency in English considered a raw machine-translated output quality lower than the post-edited text, i.e., one containing no errors. Although Hu et al.'s and our studies have different designs and purposes, it may be inferred that even non-professionals who may be expected to be ignorant of or care less about mistakes in the text are generally aware of drawbacks and notice them.

Some of our study results may also be to some extent comparable with those obtained in the investigation by Colman et al. (2021) where an increased number of eye fixations and increased gaze duration while reading machine translation segments were found in comparison with human translation (Colman et al., 2021), which may imply that less naturalistic and possibly erroneous text segments require more cognitive load. In our study, all respondents (both professionals and non-professionals) demonstrated increased values of all tested eye movement variables on areas of interest with errors compared with areas of interest without errors.

Other noteworthy findings to emerge from this study relate to the question whether there is a difference in the processing of raw machine-translated output between non-professional users with different levels of acceptability of machine-translated text. The overall acceptability of machine-translated text was found to be higher for those non-professional users who spent less time/effort on areas of interest with errors, which might be an indication that the participants who did not notice or were more positive or tolerant about the mistakes were more positive about the machine-translated text in general. The text comprehension results revealed that the subjects in the group of professional translators who scored low on the comprehension questions, demonstrated a greater number of glance counts, which may imply that the professional background may influence the level of comprehension. These findings indirectly support the more positive attitudes toward raw machine-translated text in terms of its comprehension and trust by users with lower proficiency of language as reported by Rossetti et al. (2020).

However, with a relatively small sample size, caution must be applied while interpreting the results within the group of non-professional users of machine translation as the findings might be diverse depending on the subject's background, level of education, experience, language proficiency and other variables.

## 6. Conclusions

The study was aimed at determining the acceptability of raw machine translation texts in Lithuanian, a low-resource language. An eye tracking experiment measuring acceptability *via* the comparison between professional and non-professional users of machine translation and *via* the comparisons between the respondents who assessed the quality, satisfaction with and usability of the text differently (either lower or higher than average) revealed some insightful findings. There is a difference in the machine translation output cognitive processing and acceptability between professional and non-professional users. The professional users scored better in text comprehension compared with non-professional users. One of the possible reasons for that might be the experience of professional translators in dealing with badly written (perhaps also machine-translated) text. Professional users critically assess machine translation on all components of acceptability. Non-professional users—end-users with no linguistic background—have more positive attitudes toward machine translation quality, usability and satisfaction, which may imply possible risks if machine translation is used without critical awareness, judgement and revision. The lower professional users' satisfaction with the text, and overall acceptability, may suggest that they are likely to have higher expectations for the translated text.

The major general implication of these findings is the lower awareness of non-professional users regarding the machine translation output drawbacks and imperfections, which may result in a variety of misunderstandings that might go unnoticed and ignored, as well as risks and threats with undefined consequences.

The major limitation of this study is the small and uneven sample sizes of professional and non-professional users of machine translation. More equal sample sizes of different groups would help establishing a greater degree of accuracy on this matter. Besides, the differences within the non-professionals' group should be taken into consideration, as the results may be affected by various individual characteristics of subjects. Therefore, larger controlled trials could be focused more on the differences in educational backgrounds and language proficiency of subjects as well as the provided stimulus text variety or task description to give more definitive evidence regarding acceptability of machine translation.

A further limitation concerns imperfections of eye tracking equipment. To some extent they have been mitigated, but those mitigations can also be a cause of further limitations (for example, padding AOIs by about 1 character to all sides is a common way to mitigate imprecision of eye tracking leading to failures to notice the subject looking at the AOI, but it can result in including cases when the subject is looking at the area near the AOI).

Notwithstanding these limitations, the study provides a possibility to understand more deeply the readers' cognitive processing and the level of acceptability they exhibit toward machine-translated texts. Overall, the results of the study demonstrate diversified and contrasting views of the population and call for raising public awareness and machine translation literacy improvement.

## Data availability statement

The raw data supporting the conclusions of this article will be made available by the authors, without undue reservation.

## Ethics statement

The studies involving human participants were reviewed and approved by Research Ethics Commission of Kaunas University of Technology. Written informed consent for participation was not required for this study in accordance with the national legislation and the institutional requirements.

## Author contributions

RK and JM: conceptualization. RK, JM, IP, and MP: methodology. RK, JM, IP, MP, and JH: investigation and writing—review and editing. IP and MP: data curation, visualization. RK, JM, and JH: writing—original draft preparation. All authors have read and agreed to the published version of the manuscript.

## Footnotes

^1^Approval to conduct this study was obtained from the Research Ethics Committee of Kaunas University of Technology (No. M6-2021-04 as of 2021-06-16).

## References

[B1] Carl M. Dragsted B. Elming J. Hardt D. Lykke Jakobsen A. (2011). “The process of post-editing: a pilot study,” in Copenhagen Studies in Language (Frederiksberg), 131–142.

[B2] Carl M. Gutermuth S. Hansen-Schirra S. (2015). “Chapter post-editing machine translation: efficiency, strategies, and revision processes in professional translation settings,” in Psycholinguistic and Cognitive Inquiries Into Translation and Interpreting (Amsterdam: John Benjamins Publishing Company), 145–174.

[B3] Castilho S. (2016). Measuring acceptability of machine translated enterprise content (Ph.D. thesis). Dublin: Dublin City University.

[B4] Castilho S. O'Brien S. (2016). “Evaluating the impact of light post-editing on usability,” in Proceedings of the Tenth International Conference on Language Resources and Evaluation (LREC'16) (Portoroz: European Language Resources Association, ELRA), 310–316.

[B5] Castilho S. O'Brien S. Alves F. O'Brien M. (2014). “Does post-editing increase usability? a study with Brazilian Portuguese as target language,” in Proceedings of the 17th Annual conference of the European Association for Machine Translation (Dubrovnik: European Association for Machine Translation), 183–190.

[B6] Castilho S. O'Brien S. (2018). “Acceptability of machine-translated content: a multi-language evaluation by translators and end-users,” in Linguistica Antverpiensia, New Series - Themes in Translation Studies, Vol. 16 (Antwerp).

[B7] Colman T. Fonteyne M. Daems J. Macken L. (2021). “It's all in the eyes: an eye tracking experiment to assess the readability of machine translated literature,” in 31st Meeting of Computational Linguistics in The Netherlands (CLIN 31), Abstracts (Ghent).

[B8] Daems J. Vandepitte S. Hartsuiker R. J. Macken L. (2017). Identifying the machine translation error types with the greatest impact on post-editing effort. Front. Psychol. 8, 01282. 10.3389/fpsyg.2017.01282 28824482 PMC5539081

[B9] Doherty S. (2016). Translations| the impact of translation technologies on the process and product of translation. Int. J. Commun. 10, 947–969.

[B10] Doherty S. O'Brien S. (2014). Assessing the usability of raw machine translated output: a user-centered study using eye tracking. Int. J. Hum. Comput. Interact. 30, 40–51. 10.1080/10447318.2013.802199

[B11] Fereira A Gries S. T. Schwieter J. W. (2021). “Assessing indicators of cognitive effort in professional translators: A study on language dominance and directionality,” in Translation, interpreting, cognition: The way out of the box, ed Tra&Co Group (Berlin: Language Science Press), 115–143. 10.5281/zenodo.4545041

[B12] García I. (2010). Is machine translation ready yet? Target 22, 7–21. 10.1075/target.22.1.02gar

[B13] Guerberof Arenas A. Moorkens J. O'Brien S. (2021). The impact of translation modality on user experience: an eye-tracking study of the microsoft word user interface. Mach. Transl. 35, 205–237. 10.1007/s10590-021-09267-z 34776636 PMC8550651

[B14] Hoi H. T. (2020). Machine translation and its impact in our modern society. Int. J. Sci. Technol. Res. 9, 1918–1921.

[B15] Hu K. O'Brien S. Kenny D. (2020). A reception study of machine translated subtitles for MOOCs. Perspectives 28, 521–538. 10.1080/0907676X.2019.1595069

[B16] Jakobsen A. L. Jensen K. T. H. (2008). Eye movement behaviour across four different types of reading task. Copenhagen Stud. Lang. 36, 103–124.

[B17] Kasperė R. Horbačauskienė J. Motiejunienė J. Liubinienė V. Patašienė I. Patašius M. (2021). Towards sustainable use of machine translation: usability and perceived quality from the end-user perspective. Sustainability 13, 3430. 10.3390/su132313430

[B18] Kasperavičienė R. Motiejūnienė J. Patašienė I. (2020). Quality assessment of machine translation output. Texto Livre 13, 271–285. 10.35699/1983-3652.2020.24399

[B19] Kasperė R. Motiejūnienė L. (2021). “Eye-tracking experiments in human acceptability of machine translation to study societal impacts,” in Sustainable Multilingualism 2021: The 6th International Conference, eds A. Daukšaite-Kolpakoviene and Ž. Tamašauskaite (June 4–5, 2021, Kaunas, Lithuania): book of abstracts (Kaunas: Vytautas Magnus University), 114.

[B20] Lommel A. Uszkoreit H. Burchardt A. (2014). Multidimensional quality metrics (MQM): a framework for declaring and describing translation quality metrics. Tradumàtica tecnol. trad. 12, 455. 10.5565/rev/tradumatica.77

[B21] McConkie G. W. Rayner K. (1975). The span of the effective stimulus during a fixation in reading. Percept. Psychophys. 17, 578–586. 10.3758/BF0320397228709924

[B22] Moorkens J. (2018). “Chapter Eye-Tracking as a Measure of Cognitive Effort for Post-Editing of Machine Translation,” in Eye Tracking and Multidisciplinary Studies on Translation (Amsterdam: John Benjamins Publishing Company), 55–69.

[B23] Moorkens J. O'Brien S. (2015). “Post-editing evaluations: trade-offs between novice and professional participants,” in Proceedings of the 18th Annual Conference of the European Association for Machine Translation (Antalya), 75–81.

[B24] MQM Commitee. (2022). MQM (Multidimensional Quality Metrics): what is MQM? Available online at: https://themqm.org/ (accessed October 10, 2022).

[B25] Nurminen M. Koponen M. (2020). Machine translation and fair access to information. Transl. Spaces 9, 150–169. 10.1075/ts.00025.nur

[B26] Ortega J. Sánchez-Martínez F. Turchi M. Negri M. (2019). “Improving translations by combining fuzzy-match repair with automatic post-editing,” in Proceedings of Machine Translation Summit XVII: Research Track (Dublin: European Association for Machine Translation), 256–266.

[B27] Perrault R. Shoham Y. Brynjolfsson E. Clark J. Etchemendy J. Grosz B.. (2019). The ai index 2019 annual report. Technical report, Stanford: AI Index Steering Committee, Human-Centered AI Institute, Stanford University.

[B28] Rayner K. (1998). Eye movements in reading and information processing: 20 years of research. Psychol. Bull. 124, 372–422. 10.1037/0033-2909.124.3.372 9849112

[B29] Rivera-Trigueros I. (2021). Machine translation systems and quality assessment: a systematic review. Lang. Resour. Eval. 10.1007/s10579-021-09537-534597937

[B30] Rossetti A. O'Brien S. Cadwell P. (2020). “Comprehension and trust in crises: investigating the impact of machine translation and post-editing,” in Proceedings of the 22nd Annual Conference of the European Association for Machine Translation (Lisboa: European Association for Machine Translation), 9–18. Available online at: https://aclanthology.org/2020.eamt-1.2

[B31] Rossi C. Carré A. (2022). “How to choose a suitable neural machine translation solution: Evaluation of MT quality,” in Machine Translation for Everyone: Empowering Users in the Age of Artificial Intelligence, ed D. Kenny (Berlin: Language Science Press), 51–79. 10.5281/zenodo.6759978

[B32] Rossi C. Chevrot J.-P. (2019). Uses and perceptions of machine translation at the european commission. J. Special. Transl. 31, 177–200. Available online at: https://shs.hal.science/halshs-01893120/file/Rossi_and_Chevrot_article8.pdf

[B33] Schuster M. Johnson M. Thorat N. (2016). Zero-shot Translation With Google's Multilingual Neural Machine Translation System. Google AI blog. Available online at: https://ai.googleblog.com/2016/11/zero-shot-translation-with-googles.html

[B34] Stasimioti M. Sosoni V. (2021). “Chapter Investigating post-editing: a mixed-methods study with experienced and novice translators in the English-Greek language pair, ‘ Translation, Interpreting, Cognition: The Way Out of the Box (Berlin: Language Science Press).

[B35] Taivalkoski-Shilov K. Toral A. Hadley J. L. Teixeira C. S. C. editors (2022). “Using technologies for creative-text translation,” in Routledge Advances in Translation and Interpreting Studies (London: Routledge).

[B36] Taylor R. M. Crichton N. Moult B. Gibson F. (2015). A prospective observational study of machine translation software to overcome the challenge of including ethnic diversity in healthcare research. Nurs. Open 2, 14–23. 10.1002/nop2.13 27708797 PMC5047311

[B37] Ueffing N. (2018). “Automatic post-editing and machine translation quality estimation at eBay,” in Proceedings of the AMTA 2018 Workshop on Translation Quality Estimation and Automatic Post-Editing (Boston, MA: Association for Machine Translation in the Americas), 1–34.

[B38] Vardaro J. Schaeffer M. Hansen-Schirra S. (2019). Translation quality and error recognition in professional neural machine translation post-editing. Informatics 6, 41. 10.3390/informatics6030041

[B39] Vieira L. N. (2020). Machine translation in the news. Transl. Spaces 9, 98–122. 10.1075/ts.00023.nun

[B40] Vieira L. N. O'Hagan M. O'Sullivan C. (2021). Understanding the societal impacts of machine translation: a critical review of the literature on medical and legal use cases. Inf. Commun. Soc. 24, 1515–1532. 10.1080/1369118X.2020.1776370

[B41] Yasuoka M. Bjorn P. (2011). “Machine translation effect on communication: what makes it difficult to communicate through machine translation?” in 2011 Second International Conference on Culture and Computing (Kyoto: IEEE).

